# Regulation of the general stress response sigma factor σ^T^ by Lon-mediated proteolysis

**DOI:** 10.1128/jb.00228-23

**Published:** 2023-11-06

**Authors:** Roya Akar, Matthias J. Fink, Deike J. Omnus, Kristina Jonas

**Affiliations:** 1 Science for Life Laboratory and Department of Molecular Biosciences, The Wenner-Gren Institute, Stockholm University, Svante Arrhenius, Stockholm, Sweden; Philipps-Universitat Marburg Fachbereich Biologie, Marburg, Germany

**Keywords:** regulated proteolysis, Lon protease, ECF sigma factor, general stress response, *Caulobacter crescentus*

## Abstract

The Lon protease is widely conserved in both prokaryotic and eukaryotic organisms and fulfills important regulatory functions. Nevertheless, the number of identified Lon substrates is limited in most organisms, and the precise role of Lon in regulating these proteins is poorly understood. Here, we describe the α-proteobacterial general stress response sigma factor σ^T^ as a novel Lon substrate in *Caulobacter crescentus*. Based on previously published quantitative proteomics data, we find σ^T^ to be a promising putative Lon substrate and confirm a direct role of Lon in degrading σ^T^. We show that Lon contributes to the downregulation of σ^T^ abundance under optimal conditions and during recovery from sucrose-induced osmotic stress. Furthermore, the presence of the Lon activity regulator LarA enhances Lon-mediated degradation of σ^T^ *in vitro* and reduces σ^T^ levels *in vivo* indicating a role of LarA in modulating Lon-mediated degradation of σ^T^. Together, our results highlight the importance of Lon during the recovery phase following stress exposure by adjusting the concentrations of critical regulators of stress responses.

**IMPORTANCE:**

Regulated protein degradation is a critical process in all cell types, which contributes to the precise regulation of protein amounts in response to internal and external cues. In bacteria, protein degradation is carried out by ATP-dependent proteases. Although past work revealed detailed insights into the operation principles of these proteases, there is limited knowledge about the substrate proteins that are degraded by distinct proteases and the regulatory role of proteolysis in cellular processes. This study reveals a direct role of the conserved protease Lon in regulating σ^T^, a transcriptional regulator of the general stress response in α-proteobacteria. Our work is significant as it underscores the importance of regulated proteolysis in modulating the levels of key regulatory proteins under changing conditions.

## INTRODUCTION

The proteome of all living cells is highly dynamic and changes in its composition in response to various internal and external cues. In order to maintain cellular integrity and to adapt to changing conditions, cells constantly synthesize new proteins and degrade others. Proteolysis plays an important role in removing damaged and non-functional proteins that can accumulate as a consequence of mistranslation or proteotoxic stress. Additionally, proteolysis constitutes an important regulatory mechanism that complements transcriptional and translational mechanisms by precisely adjusting the concentrations of specific functional proteins. In many cases, proteolysis has been shown to be critical for the rapid reduction in protein levels in response to distinct growth conditions and environmental signals (1). This can be particularly important during different stages of developmental processes or during the recovery phase following acute stress, when stress-induced proteins are no longer needed and have to be removed to allow resumption of normal growth (2, 3).

Proteolysis is carried out by dedicated proteases. The major proteases in prokaryotes belong to the ATPases Associated with diverse cellular Activities (AAA+) protease family and one widely conserved member of the family is the protease Lon. Like other AAA+ proteases, Lon forms a barrel-shaped complex consisting of an ATPase and a peptidase domain that unfold, translocate, and proteolytically digest protein substrates (4, 5). Additionally, Lon possesses an extended N-terminal domain that is flexibly linked to the ATPase module and is critical for substrate recognition (4). Most substrates are recognized via short peptide sequences called degradation tags or degrons that are often rich in aromatic and hydrophobic residues and in many cases located at the C- or N-terminus of the substrate protein (6). Despite increasing insights into the degron sequences facilitating Lon recognition, it is still challenging to predict protease substrates based on sequence information and the identification of specific substrates relies on experimental procedures.

*Caulobacter crescentus* is an important model for bacterial cell biology (7). This organism has a dimorphic life cycle, in which each cell division results in distinct daughter cells, a motile swarmer cell, and a sessile stalked cell. While the stalked cell is capable of DNA replication and cell division, the swarmer cell is G1-arrested and must differentiate into a stalked cell before entering S-phase. Progression through the *C. crescentus* cell cycle depends on large-scale changes of the proteome composition to ensure that morphological transitions are coordinated with key cell cycle events (8). A large body of previous work revealed the importance of tightly controlled gene expression and proteolysis in this process (8, 9). More recent work has also elucidated a role of Lon in temporally regulating the abundances of important regulatory and structural proteins during the cell cycle (10 – 13).

As a freshwater bacterium that is frequently exposed to environmental fluctuations, *C. crescentus* needs to regulate the composition of its proteome also in response to environmental inputs. Like other bacteria, *C. crescentus* makes use of multiple subunits of RNA polymerase (RNAP) to globally reprogram transcription in response to specific growth conditions (2, 14). Under optimal conditions, RNAP associates with the housekeeping sigma factor σ^70^ that drives the expression of a large group of genes needed for growth and proliferation. Specific stress conditions induce the expression and activation of alternative sigma factors that can replace σ^70^ and direct RNAP to the promoters of genes needed for stress adaptation (14). In *C. crescentus*, σ^T^ (encoded by the *sigT* gene) was described as a key regulator of the general stress response that is upregulated under a range of stresses, including osmotic and oxidative stress, and regulates a group of genes to ensure survival under such conditions (15, 16). As a member of the extracytoplasmic function (ECF) family of sigma factors, σ^T^ is expressed with its cognate anti-sigma factor NepR that binds σ^T^ and sequesters it away from the RNA polymerase complex under optimal conditions (17). At the onset of stress, σ^T^ is liberated from NepR by phosphorylated PhyR through a partner-switching mechanism (17 – 19), which then allows σ^T^ to bind to RNAP and drive expression of σ^T^-regulated promoters. Since σ^T^ positively regulates its own transcription, *sigT* expression is induced at the onset of stress (15). As stress ceases, the expression of σ^T^-regulated genes needs to be shut off again, and σ^T^ abundance and activity reset to pre-stress levels. However, how σ^T^ levels are regulated during stress recovery is currently not fully understood.

A previous study led to the proteomics-based identification of novel Lon substrates in *C. crescentus* (10). While this previous work focused on the detailed characterization of novel Lon substrates involved in *C. crescentus* development (10), the precise role of Lon in degrading other putative substrates remains to be studied. Here, we focus on the role of Lon in regulating the general stress response regulator σ^T^. We show that σ^T^ is a direct target of Lon-mediated proteolysis and that Lon contributes to the downregulation of σ^T^ levels under optimal conditions and in the recovery phase after acute sucrose stress. Furthermore, we show that the Lon activity regulator LarA enhances degradation of σ^T^ *in vitro* and that σ^T^ levels respond to overexpression of LarA *in vivo* indicating that LarA might be involved in regulating the degradation of σ^T^ under certain conditions.

## RESULTS

### The ECF sigma factor σ^T^ is a promising putative Lon substrate

**Fig 1:**
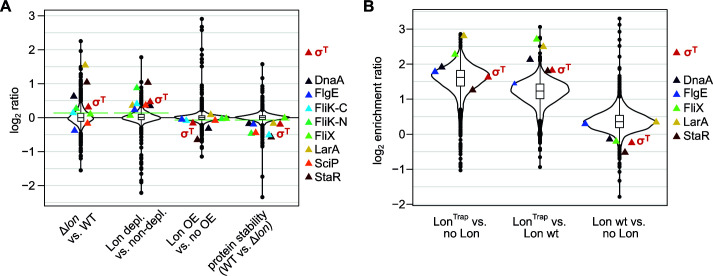
Proteomics data indicate that σ^T^ is a putative Lon substrate. (A) Violin plots showing proteome-wide changes in protein abundances (log_2_ ratios) for the indicated strain comparisons (Lon depl, Lon depletion; OE, overexpression). The plots are based on quantitative proteomics data published in reference (10). Data are shown as distributions and the median and quartiles using an overlayed box plot. Colored data points represent log_2_ ratios of σ^T^ and previously identified substrates of Lon. The green lines indicate the cut-offs used in reference (10) to identify putative Lon substrates. (B) Violin plots showing distributions of log_2_ enrichment ratios for proteins co-purified with a strep-tagged Lon or Lon^Trap^ compared either to each other or to no Lon control. The no-Lon control corresponds to processed sample of cells not expressing Lon. Data are shown as distributions and the median and quartiles using an overlayed box plot. Colored data points represent log_2_ ratios of σ^T^ and previously identified substrates of Lon. The plots are based on mass spectrometry (MS)-based proteomics data provided in reference (20).

A previous quantitative proteomics study, in which proteome-wide changes in protein abundances and stability were determined in cells that either lacked or overproduced Lon, led to the identification of 146 potential Lon substrates in *C. crescentus* (10). Inspecting the list of putative Lon substrates revealed the σ^T^ protein as a promising candidate. According to the published proteomics data, σ^T^ showed Lon-dependent changes in protein levels that are similar to other previously described substrates of Lon (Fig. 1A). σ^T^ belonged to the most upregulated proteins in a strain lacking Lon (Δ*lon*) compared to the wild type and was also upregulated in a Lon depletion strain under depleting conditions compared to non-depleting conditions. In contrast, *lon* overexpression mildly reduced the abundance of σ^T^ (Fig. 1A). Furthermore, σ^T^ was among the proteins that were stabilized following a protein synthesis shut-off in Δ*lon* cells (Fig. 1A).

Consistent with the hypothesis that σ^T^ is a Lon substrate, σ^T^ also belonged to the most strongly enriched proteins in a previous pull-down experiment utilizing a proteolytically inactive Lon variant (Lon^Trap^), which was aimed at the identification of direct Lon interactors (20). Specifically, co-purified σ^T^ was enriched in the elution fraction from cells expressing Lon^Trap^ compared to cells that either lacked Lon (Fig. 1B) or that expressed the wild-type version of Lon (Fig. 1B). Conversely, σ^T^ was less abundant in the elution fraction from Lon wild-type expressing cells compared to the no-Lon control (Fig. 1B). As in the case of the quantitative proteomics experiment, σ^T^ behaved in this pull-down experiment similarly to previously confirmed substrates of Lon but differently from the bulk of proteins, again suggesting that σ^T^ is a Lon substrate.

### Lon degrades σ^T^ *in vitro*

**Fig 2:**
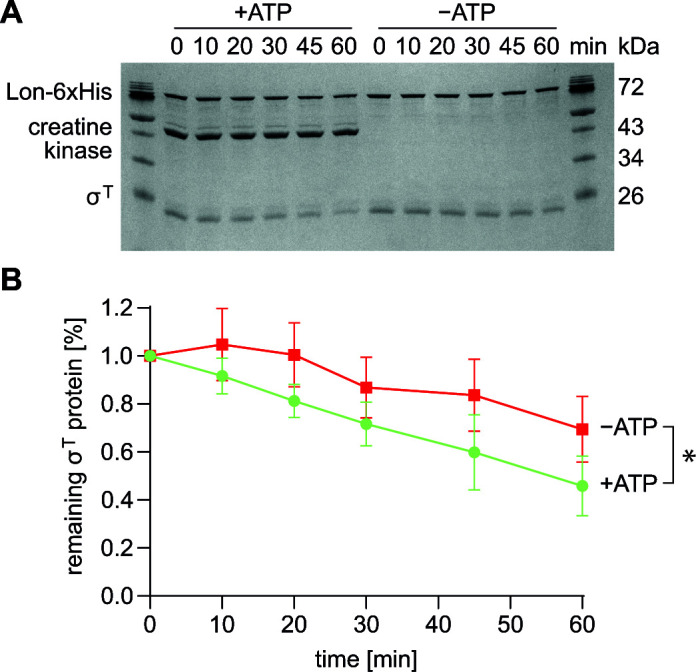
Lon degrades σ^T^ *in vitro*. (**A**) SDS-PAGE showing *in vitro* degradation of σ^T^ by Lon either in the presence or in the absence of a creatine kinase-dependent ATP regeneration system. The assay contained 0.125 µM purified, hexameric Lon-His and 2 µM purified σ^T^ in the presence of a creatine kinase-dependent ATP regeneration system, and samples were taken at the indicated time points. (**B**) Quantifications of band intensities of the assay shown in A. Data points represent means of four separate reactions, and error bars represent standard deviations. Statistical analysis between both conditions using a simple linear regression analysis in GraphPad Prism showed that the slopes of the fitted lines (+ATP 0.0008962 vs –ATP 0.001241) differ significantly (*P* = 0.0309).

To experimentally test if σ^T^ is a Lon substrate, we purified σ^T^ and monitored its degradation by purified Lon in an *in vitro* degradation assay using a creatine kinase-based ATP regeneration system. In the presence of ATP and Lon, σ^T^ levels steadily decreased with increasing incubation time, leading to a 45% reduction in protein levels after 60 min of incubation (Fig. 2A and B). Notably, σ^T^ levels also decreased to some degree in reactions lacking either ATP or Lon (Fig. 2A and B; Fig. S1). However, since σ^T^ clearance was significantly faster in the +ATP reaction than in the −ATP reaction, we conclude that σ^T^ is degraded by Lon in an ATP-dependent manner, thus validating that σ^T^ is a Lon substrate. Since the rate of σ^T^ degradation was relatively low compared to some other substrates of Lon [e.g., SciP (11) or LarA (20)], other factors might be needed that promote efficient Lon-dependent degradation of σ^T^.

### Lon contributes to σ^T^ downregulation during optimal growth and stress recovery

**Fig 3:**
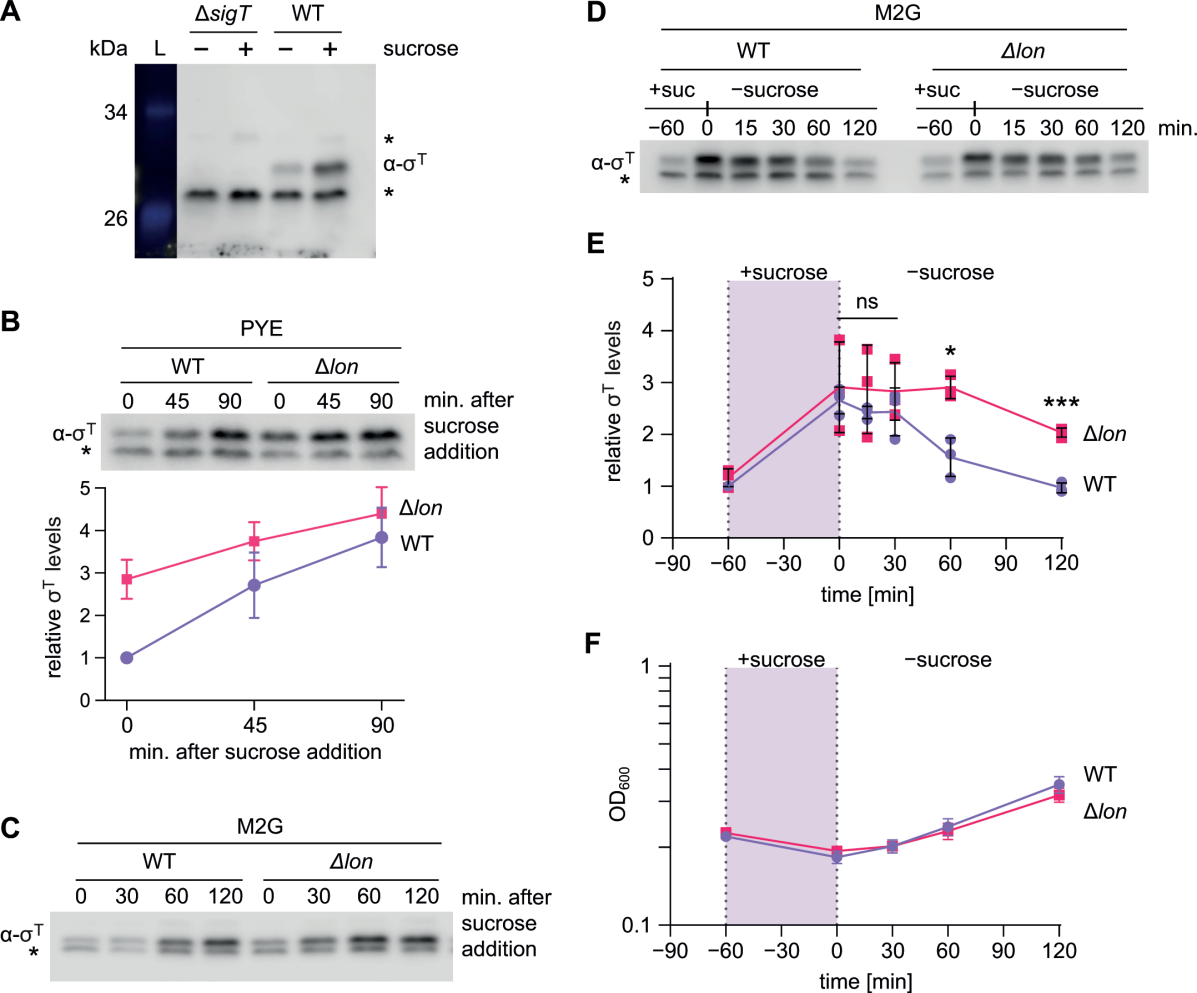
Downregulation of σ^T^ levels during stress recovery is delayed in Δ*lon* cells. (**A**) Immunoblot analysis validating the specificity of an anti-σ^T^ antibody. Cell lysates were prepared from *C. crescentus* wild type (WT) and Δ*sigT* cells that were grown either in the absence of sucrose (−) or for 1 h in the presence of 150 mM sucrose (+). σ^T^ and two non-σ^T^ bands (*) are detected by the antibody. A colorimetric image of the lane containing the NEB broad range color prestained protein standard showing the relevant bands was added for reference. (**B**) Immunoblot analysis of σ^T^ abundance in *C. crescentus* wild type (WT) and Δ*lon* cells before (*t* = 0 min) and after 45 and 90 min of exposure to 150 mM sucrose when grown in PYE medium. Quantifications below the immunoblot display means ± SD of four biological replicates. (**C**) Immunoblot analysis of σ^T^ abundance in *C. crescentus* WT and Δ*lon* cells before (*t* = 0 min) and after 30, 60, and 120 min of exposure to 150 mM sucrose when grown in M2G medium. (**D**) Abundance of σ^T^ in WT and Δ*lon* cells during stress exposure and stress recovery. Cultures were pre-treated for 60 min with 150 mM sucrose to induce stress and then allowed to recover in fresh M2G for 120 min. Protein abundances were measured before the pre-treatment (−60 min) and after 0, 15, 30, 60, and 120 min of recovery. The *t* = 0 sample was taken just before cells were moved from the sucrose stress condition to fresh M2G medium and thus corresponds to 60 min of sucrose stress. (**E**) Quantifications of band intensities of the immunoblot analysis shown in C. Data points represent the means ± SD from three biological replicates and were quantified relative to pre-stress σ^T^ levels in WT cells. Values for time points ≥ 0 were compared using a Welch *t*-test. *P < 0.05; **P < 0.01; ***P < 0.001; ****P < 0.0001; ns, not significant. (**F**) Growth kinetics WT and Δ*lon* cells during stress exposure and stress recovery as measured by changes in OD_600_ over the indicated time points. Data are shown as means ± SD.

Next, we wanted to investigate the relationship between σ^T^ and Lon *in vivo*. According to previous studies, the *sigT* gene is expressed at low levels during growth in the complex medium PYE and at somewhat higher levels in the glucose-supplemented minimal medium M2G (15). At the onset of various stress conditions, including hyperosmolarity induced by sodium chloride or sucrose, oxidative stress, carbon starvation, and temperature upshifts, *sigT* expression is strongly induced (15, 21). To quantify σ^T^ protein levels *in vivo*, we raised σ^T^-specific antiserum. Immunoblot analysis with this antiserum yielded one faint band at around 32 kDa and two stronger bands at 30 and 28 kDa in the wild type (Fig. 3A). The 30-kDa band increased in intensity during the sucrose treatment and was absent in a Δ*sigT* mutant, indicating that it corresponds to σ^T^. To assess the role of Lon in regulating σ^T^ abundance, we compared σ^T^ levels between wild-type and Δ*lon* cells before and after exposure to sucrose stress (Fig. 3B). We focused on sucrose stress since σ^T^ is most strongly induced and required for survival under this condition, according to previous work (15). Consistent with these data, we found that σ^T^ protein clearly accumulated over the course of sucrose treatment (Fig. 3B and C). When grown in the complex medium PYE, we found that σ^T^ levels were approximately twofold to threefold higher in the Δ*lon* strain compared to the wild type before sucrose addition (Fig. 3B). This result is consistent with the proteomics data (Fig. 1A) and indicates that Lon contributes to the maintenance of low σ^T^ levels in the absence of stress. After sucrose addition, σ^T^ levels accumulated in the Δ*lon* mutant to similar levels as in the wild type, suggesting that the stress-dependent upregulation of σ^T^ level is largely due to increased *sigT* expression.

Interestingly, we did not observe a significant difference in σ^T^ levels between the wild-type and Δ*lon* strains, when cultured in M2G medium (Fig. 3C). We reasoned that Lon-dependent proteolysis might play a role in the recovery phase after sucrose stress treatment. To test this idea, we monitored steady-state levels of σ^T^ in WT and Δ*lon* cells that were first exposed to sucrose stress for 1 h and subsequently shifted to M2G medium without sucrose and allowed to recover for 2 h. Consistent with our other data, σ^T^ levels increased in the presence of sucrose in both strains to approximately similar levels (Fig. 3D and E). Interestingly, during the stress recovery phase, the two strains showed different kinetics in σ^T^ downregulation. In the wild type, σ^T^ levels started to decrease 15–30 min after shifting the culture back to non-stress conditions and reached pre-stress levels after 2 h of recovery (Fig. 3D and E). In contrast, σ^T^ levels in the Δ*lon* strains remained high until approximately 1 h after the shift to non-stress conditions and failed to reach pre-stress levels within 2 h (Fig. 3D and E). Both strains showed similar growth during the recovery phase (Fig. 3F), making it unlikely that growth-dependent indirect effects caused the delay in σ^T^ downregulation in the Δ*lon* mutant. These results indicate that Lon-dependent proteolysis contributes to the downregulation of σ^T^ to pre-stress levels during the recovery phase following sucrose-induced stress.

### The Lon regulator LarA enhances Lon-mediated σ^T^ degradation *in vitro*

**Fig 4:**
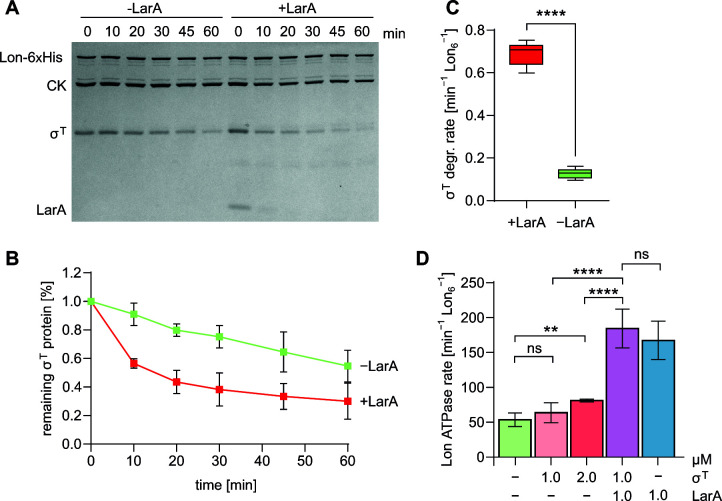
Lon-mediated degradation of σ^T^ is enhanced by LarA. (**A**) SDS-PAGE showing Lon-mediated σ^T^ degradation in the presence and absence of LarA. Two-micromolar σ^T^ and 0.125 µM hexameric Lon-His were used either without (−LarA) or with 4 µM LarA (+LarA). A creatine kinase (CK)-dependent ATP regeneration system was added to all reactions. (**B**) Quantifications of band intensities of the assay shown in A. Means ± SD were determined based on six independent reactions. (**C**) Degradation rates of σ^T^ by Lon-His in the presence and absence of LarA. Degradation rates were calculated by fitting a linear regression either to the values of each reaction shown in B (−LarA condition) or to values from reactions sampled every 2 min for 10 min (+LarA condition). Statistical significance was determined using an unpaired *t*-test: ****P < 0.0001. (**D**) ATPase rates of Lon-His hexamer (0.05 µM) without substrate (−) or in the presence of σ^T^, LarA, or a combination of σ^T^ and LarA at the indicated concentration. Values represent means ± SD of at least three independent measurements. The data for Lon without substrate (−) include data from experimental replicates that were published in reference (20). Statistical significance was determined by performing pairwise two-sided, unpaired *t*-tests with subsequent Holm correction of the *P*-values. *P < 0.05; **P < 0.01; ***P < 0.001; ****P < 0.0001; ns, not significant.

The activity of the AAA+ Lon protease in *C. crescentus* is regulated by a recently discovered heat shock protein, LarA, which is a Lon substrate itself (20). LarA is an allosteric regulator of Lon activity that modulates degradation rates of a range of Lon substrates (20). As LarA and σ^T^ are both stress-induced in *C. crescentus* and because degradation of σ^T^ by Lon was relatively slow in the absence of additional factors (Fig. 2), we wondered if LarA affects σ^T^ proteolysis by Lon and measured σ^T^ degradation *in vitro* either in the absence or in the presence of LarA. Indeed, addition of LarA to the reaction noticeably enhanced σ^T^ degradation. The enhancing effect of LarA was particularly strong during the first 10 min of the assay, before LarA was eliminated from the reaction due to its own degradation by Lon (Fig. 4A and B). Calculation of σ^T^ degradation rates in the absence and presence of LarA showed that the σ^T^ degradation rate significantly increased by about 5.4-fold from 0.128 ± 0.022 without LarA to 0.690 ± 0.055 min^−1^ Lon_6_^−1^ in the presence of LarA (Fig. 4C).

We also analyzed the effect of σ^T^ on the ATPase activity of Lon. Incubation of Lon in the presence of 1 µM of σ^T^ did not notably increase the ATPase rate of Lon compared to a reaction containing Lon alone (Fig. 4D). However, addition of 2 µM of σ^T^ led to a mild but significant increase of Lon’s ATPase activity (Fig. 4D). Simultaneous incubation of Lon with 1 µM σ^T^ and 1 µM LarA led to a strong increase in Lon’s ATPase activity. Although the resulting ATPase rate was comparable to the ATPase rate of Lon when 1 µM LarA was present as the only substrate, this result indicates that the fast degradation of σ^T^ in the presence of LarA correlates with a high ATPase activity of Lon. As previously proposed (20), this could mean that LarA acts on Lon by stabilizing Lon in a conformation with high ATPase activity.

### LarA overexpression reduces σ^T^ levels *in vivo*

**Fig 5:**
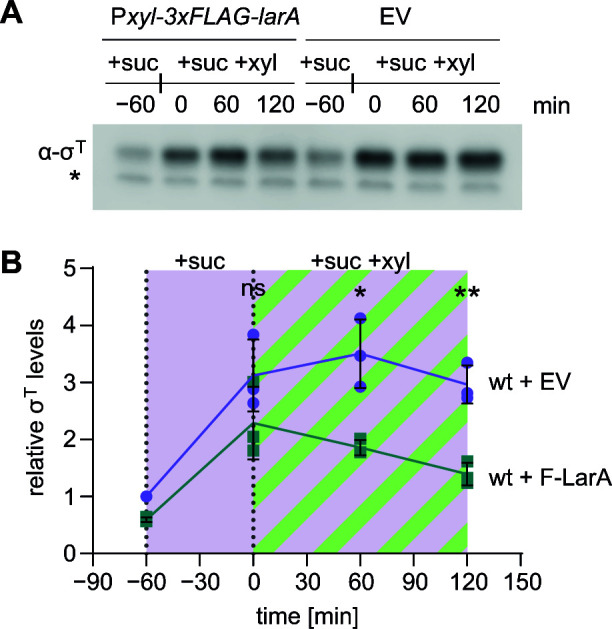
Overexpression of LarA reduces cellular σ^T^ levels during sucrose stress. (**A**) Immunoblot analysis of *C. crescentus* wild-type cells carrying either a vector with xylose-inducible *larA* (*P_xyl_-3xFLAG-larA*) or an empty vector (EV). Cells were first exposed to 150 mM sucrose for 60 min (+suc) before additionally inducing *larA* expression with xylose (+suc +xyl). σ^T^ protein levels were analyzed 60 min before xylose addition (−60) and 0, 60, and 120 min after xylose addition. (**B**) Quantifications of band intensities from three independent replicates of the immunoblot shown in A. Data points represent means ± SD. Values for time points ≥ 0 were compared using a Welch *t*-test. *P < 0.05; **P < 0.01; ***P < 0.001; ****P < 0.0001; ns, not significant.

In order to investigate whether LarA enhances σ^T^ degradation *in vivo*, we determined the effect of *larA* overexpression on the steady-state levels of σ^T^. Exponentially growing wild-type cells containing either a plasmid harboring a xylose-inducible copy of *larA* (*P_xyl_-3×FLAG-larA*) or an empty plasmid were first exposed to 150 mM sucrose for 1 h before FLAG-LarA (F-LarA) expression from the plasmid was induced by addition of xylose (Fig. 5A). For both strains, an upregulation of σ^T^ could be observed following stress exposure (Fig. 5A and B). However, in the F-LarA overexpression, strain σ^T^ levels were at all time points clearly lower compared to the vector control strain. σ^T^ levels were reduced even under non-inducing conditions, which can likely be explained by leaky expression of F-LarA from the *P_xyl_* promoter. After induction with xylose, the difference between the two strains further increased (Fig. 5A and B). While σ^T^ levels in the empty vector control remained at a high level, σ^T^ levels in the overexpression strain decreased continuously within 2 h. Similar to these results that we obtained with cultures grown in M2G, we also observed a repressing effect of F-LarA on σ^T^ levels in sucrose-treated cells grown in PYE (Fig. S2). Together, these data show that the presence of LarA reduces σ^T^ levels *in vivo*. This result is consistent with the LarA-dependent activation of σ^T^ degradation that we have observed *in vitro* and suggests a regulatory role of LarA in σ^T^ degradation.

## DISCUSSION

Intracellular proteolysis is a critical process in all cells, which impacts the concentrations of key regulatory proteins involved in various cellular processes, including cell differentiation and stress responses. In the dimorphic *C. crescentus*, the protease Lon has been linked to the heat shock response (22) and the responses to oxygen-limiting and DNA damage-inducing growth conditions (23, 24). Here, we show that Lon is also involved in the general stress response by directly regulating the ECF sigma factor σ^T^.

σ^T^ was previously described as the main regulator of the general stress response in *C. crescentus* and other α-proteobacteria (16). With its critical roles in stress survival and adaptation, this protein must be precisely regulated in response to environmental conditions, both on the level of protein activity and on the level of protein abundance. Our new work revealed that Lon-mediated proteolysis represents an additional layer of σ^T^ regulation. σ^T^ was among the most promising hits in two independent proteomics searches for Lon substrates (10, 20). Consistent with these data, we found that Lon specifically degrades σ^T^. Although σ^T^ degradation *in vitro* was relatively slow when no additional factors were added to the reaction, σ^T^ degradation was highly efficient in the presence of LarA, a recently discovered regulator of Lon activity (20). The expression of *larA* is stress induced itself (20). As a σ^32^-regulated heat shock protein, it is strongly induced at the onset of proteotoxic stress conditions (20), and previous RNA sequencing experiments indicate that its expression is also upregulated during salt-induced osmotic stress (25). Hence, LarA may modulate σ^T^ degradation under specific stress conditions.

**Fig 6:**
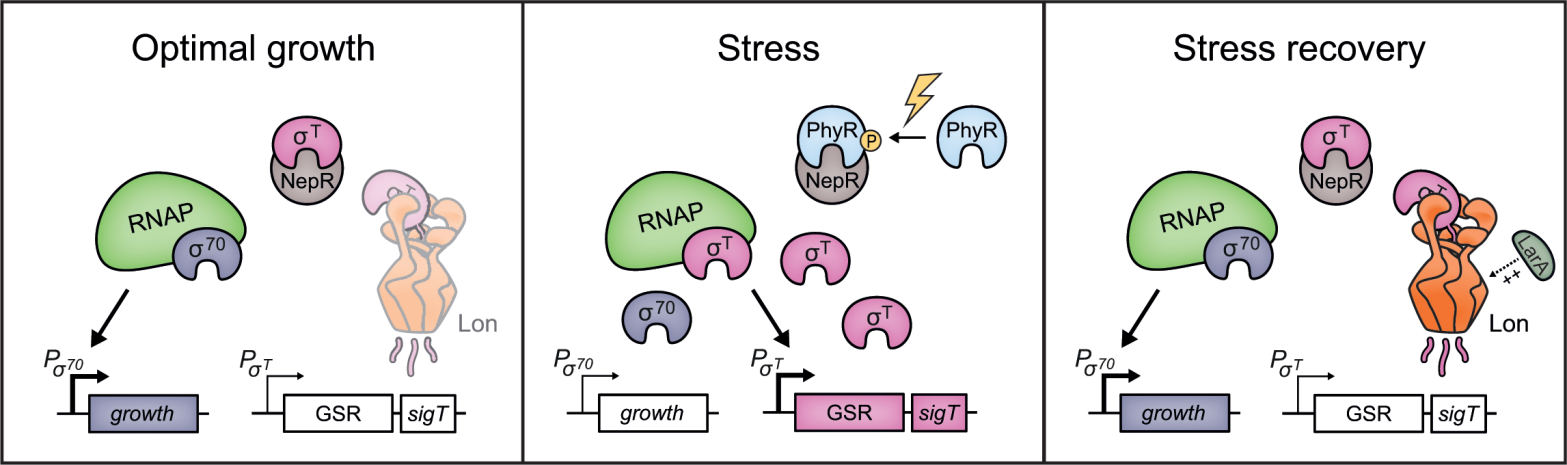
Model of Lon-mediated proteolysis of σ^T^ during stress recovery. Under optimal conditions, σ^T^ is present at low levels and bound by its anti-sigma factor NepR leading to the repression of the general stress response (GSR) (16, 17). Lon proteolysis contributes to maintaining low σ^T^ levels, at least in the complex medium PYE. At the onset of stress, σ^T^ is released from NepR, binds RNAP, and induces the GSR, which also induces its own expression (15 – 17). During stress recovery, Lon proteolysis helps to reset σ^T^ abundance to pre-stress levels, which may be promoted by LarA. σ^70^ will outcompete σ^T^ for binding to RNAP, leading to the activation of σ^70^-controlled genes and consequently growth resumption.

Our data show that Lon helps to maintain low σ^T^ abundance under optimal conditions when cells grow in the rich medium PYE (Fig. 6). Additionally, we found that the downregulation of σ^T^ in the stress recovery phase after sucrose-induced osmotic stress was delayed in the Δ*lon* strain. This result suggests that Lon contributes to the downregulation of σ^T^ abundance particularly in the stress recovery phase, when *sigT* expression ceases. Downregulating σ^T^ levels likely works in concert with NepR-dependent sequestration of σ^T^ to reduce the amount of available σ^T^ (Fig. 6). Reduced σ^T^ availability will allow the housekeeping sigma factor σ^70^ to outcompete σ^T^ for binding to RNAP, thus allowing cells to re-allocate their resources to proliferative functions, while turning off the expression of σ^T^-dependent stress genes.

While our data show that Lon helps to adjust σ^T^ under optimal conditions and during stress recovery, Lon did not notably affect σ^T^ abundance during sucrose-induced osmotic stress. Degradation of σ^T^ at the onset of stress would counteract the stress-induced accumulation of σ^T^. Thus, a low rate or absence of σ^T^ degradation upon stress exposure likely enables rapid upregulation of σ^T^ protein levels, when σ^T^ function is needed.

In addition to LarA, other factors might influence σ^T^ degradation by Lon. For example, binding of σ^T^ to NepR could affect the accessibility of the degron of σ^T^. This interaction may either hide the degron of σ^T^ rendering the σ^T^–NepR complex more stable than σ^T^ alone or, in an alternative scenario, the σ^T^–NepR complex might reveal a binding site or degron allowing more efficient degradation of σ^T^. Furthermore, Lon-dependent degradation of σ^T^ could be affected by DNA binding. In *C. crescentus*, Lon was suggested to degrade DNA-bound proteins under DNA damage stress to facilitate DNA repair (23). In another example, binding of the Lon substrate SciP to DNA and its binding partner CtrA was shown to protect SciP from efficient Lon-mediated proteolysis (11). Similarly, σ^T^ degradation might be affected, either positively or negatively, by σ^T^ binding to DNA and/or RNAP.

In conclusion, our work highlights the role of Lon in removing σ^T^ during the recovery phase following acute stress, which may help cells to reallocate their resources from protective to proliferative functions. A similar role of Lon has been suggested during the recovery phase from DNA damage stress in *E. coli*, when Lon contributes to the resumption of normal growth by degrading the SOS-induced protein SulA (26), an inhibitor of cell division under DNA damage stress (27). It is likely that Lon enables the rapid clearance of many other stress-induced regulatory proteins during the stress recovery phase in *C. crescentus* as well as other bacteria.

## MATERIALS AND METHODS

### Standard growth conditions

All bacterial strains used in this study are listed in Table S1. *C. crescentus* were cultured in PYE or the minimal medium M2G while shaking (200 rpm) at 30°C. Plasmid-carrying strains were grown in the presence of 5 µg/mL gentamycin. Sucrose stress treatment was induced by addition of 150 mM sucrose to exponentially growing cultures (OD_600_ 0.2). In stress recovery experiments, cells were shifted from sucrose-containing media to fresh M2G media by pelleting the cells at 7,197 × *g* for 10 min at room temperature, removing the medium and resuspending the cells in fresh M2G without sucrose. In the *larA* overexpression experiment, 3% xylose was added to the growing cultures to activate the *P_xyl_* promoter.

*E. coli* Dh5α‍ was used for cloning purposes and grown at 37°C in standard lysogeny broth (LB) or LB agar supplemented with 50/30 µg/mL kanamycin (solid/liquid). *E. coli* BL21-SI was grown using media lacking salt. For growth on plates, LBON agar supplemented with 50 µg/mL kanamycin and 40 µg/mL chloramphenicol was used. Liquid cultures were grown in 2xYTON supplemented with 30 µg/mL kanamycin and 20 µg/mL chloramphenicol.

### Plasmid construction

All plasmids used in this study are listed in Table S2. The expression plasmid pMF59 was created as described in reference (10). The σ^T^ coding sequence used as an insert was amplified with Phusion DNA polymerase (NEB; M0530) from genomic DNA using the following primers: AGAGAACAGATTGGTGGGATGGTCGCGGAACAG and GGAGCTCGAATTCGGATCGTAAAGACGGTCACCGC.

### σ^T^ purification

σ^T^ purification was done according to a previously published procedure (10). BL21- SI/pCodonPlus cells were transformed by electroporation using a pSUMO-YHRC derived vector. Transformed single colonies were selected from an LB agar plate lacking salt (LBON) supplemented with kanamycin and chloramphenicol antibiotics. The pre-cultures were inoculated with approximately 20 single colonies in 2xYTON medium and grown at 30°C overnight. Pre-cultures were diluted 1:100 in 1-L 2xYTON and grown to an OD_600_ of 1.0–1.5. To the main cultures, 0.5 mM IPTG and 0.3 M NaCl were added to induce the protein expression overnight at a low temperature, 20°C. Culture cell pellets were acquired by centrifugation at 6,900 × *g* for 10 min and then were stored dry at a −80°C freezer. For lysis of the cells, the pellets were resuspended in approximately 30 mL HK500MG (40 mM HEPES-KOH pH 7.5, 500 mM KCl, 5 mM MgCl, and 5% glycerol). To the cell suspensions, 1 mM PMSF, 1 mg/mL lysozyme, and 9 µL benzonase were added, and the cells were run through the EmulsiFlex-C3 high-pressure homogenizer two or three times. The lysed cells were collected and centrifuged at 32,500 × *g* at 4°C for 1 h to separate the cell debris and obtain the cleared lysates. The 6× His-SUMO-tagged σ^T^ was bound to 3 g Talon beads on ice and frequently swirled for 30 min. Talon beads were then washed five times with approximately 45 mL of HK500MG buffer; the protein was then eluted in fractions using HK500MG buffer with the addition of 250 mM Imidazole. The fractions with protein concentrations ≥ 1 mg/mL were pooled, and 1/100 volume of 4 mµg/mL Ulp1-6xHis was added to cleave and remove the 6xHis-SUMO tag from σ^T^. The imidazole was removed from the protein buffer by overnight dialysis against HK500MG. Following day, σ^T^ was separated from the tag by binding the 6xHis-SUMO tag to 3 g Talon beads. The flow-through that contained the untagged, purified σ^T^ was collected. The purity and concentration of σ^T^ were determined by SDS-PAGE (Bio-Rad 4%–20% Mini-PROTEAN TGX Stain-Free Protein Gel) stained with InstantBlue Protein Stain overnight and was imaged and quantified using Bio-Rad ImageLab program.

### σ^T^ refolding

After the purification, only a small fraction of σ^T^ was in the soluble fraction, while a considerable amount of the purified σ^T^ precipitated after cleavage of the His-SUMO tag. Therefore, protein refolding was carried out according to a previous study (10) with some adjustments to the buffer. First, the precipitated σ^T^ was isolated from the soluble fraction by centrifugation at 7,197 × *g* at 4°C for 10 min. Precipitated σ^T^ was solubilized in buffer S (50 mM HEPES pH 8.0, 6 M guanidinium-HCl, 1 mM EDTA, and 10 mM DTT), and the protein concentration was adjusted to 0.2 mg/mL before staring the refolding process. The concentration of guanidinium hydrochloride was reduced successively via stepwise dialysis, each carried out overnight at 4°C. First dialysis was performed against 125 vol dialysis buffer D1 (50 mM HEPES pH 8.0, 2 M guanidinium-HCl, and 1 mM EDTA) followed by 125 vol dialysis buffer D2 (50 mM HEPES pH 8.0, 1 M guanidinium-HCl, 1 mM EDTA, 0.4 M sucrose, 500 mM KCl, and 2 mM DTT). For the third dialysis, buffer D2 was diluted with an equal volume buffer D3 (50 mM HEPES pH 8.0, 1 mM EDTA, 0.4 M Sucrose, 500 mM KCl, and 2 mM DTT) and dialyzed overnight at 4°C. Afterwards, the protein solution was dialysed against 125 vol dialysis buffer D3. After recovering the σ^T^ solution from the dialysis membrane, it was centrifuged at 20,000 × *g* at 4°C for 10 min to remove remaining precipitate. The soluble, refolded σ^T^ was collected and supplemented with 2 mM DTT. The protein was then concentrated by centrifugation at 5,000 × *g* for 10 min at 4°C with a centrifugal filter with a molecular weight cutoff of 3 kDa. Afterwards, aliquots were snap frozen in liquid N_2_ and stored at −80°C. The final concentration was determined by SDS-PAGE using a BSA standard and visualized with InstantBlue Protein Stain (Sigma-Aldrich) or ReadyBlue Protein Stain (Sigma-Aldrich) and the gel was imaged and quantified using Bio-Rad ImageLab 6.0.1.

### σ^T^ antiserum production

Purified σ^T^ was used as an antigen to generate polyclonal antibodies raised in rabbits. The purified protein was loaded on a single-well 4%–20% Mini-PROTEAN TGX gel (Bio-Rad #4561091); the specific band corresponding to σ^T^ was cut from the gel and sent dry at ambient temperature to Davids Biotechnologie GmbH for immunization of a single rabbit. The antibody production included subsequent purification of the antiserum via affinity purification using resin-bound the σ^T^.

### *In vitro* degradation assay

Lon-dependent *in vitro* degradation assays were done as in reference (10). Proteins were separated via SDS-PAGE on 4%–20% Mini-PROTEAN TGX gel and visualized with either InstantBlue (Sigma-Aldrich) or ReadyBlue (Sigma-Aldrich). Quantifications were done in Bio-Rad ImageLab 6.0.1. Protein levels of the substrates were normalized to Lon levels of the respective band. Degradation rates in Fig. 4C were determined using the linest formula in Microsoft Excel. Either the first two data points (+LarA) or data points over the full range (−LarA) were used. Resulting values were plotted and analyzed using GraphPad Prism (v9.5.1).

### ATPase assay

The ATPase assays were conducted following the same procedures as described in detail by Omnus et al. (20). In brief, the rates are determined by measuring oxidation of NADH to NAD^+^ by lactate dehydrogenase that oxidizes pyruvate. The reactions were carried out in Lon reaction buffer (see *in vitro* degradation assays) including 10% glycerol, and measurements were done with a Spark microplate reader (Tecan). The analysis of the raw measurements was carried out in RStudio (https://www.rstudio.com) using R version 4.2.0, as previously described.

### Immunoblot analysis

Samples for immunoblot analysis were spun at 13,000 rpm for 3 min. After removal of the supernatant, the dry pellet was snap frozen in liquid nitrogen until further processing. At the end of the experiment, the pellet was resuspended in 20 µL 1× Laemmli sample buffer per OD_600_ of 0.1 and boiled at 98°C for 10 min. SDS-PAGE of the samples was performed on stain‐free 12% Mini-PROTEAN Stain-Free Protein Gels and later transferred to nitrocellulose membranes by a standard Western blot procedure using a Trans-blot turbo transfer system from Bio-Rad. The membrane was blocked overnight with 10% or 5% milk powder in Tris-buffered saline (TBS). Subsequently, the membrane was incubated overnight at 4°C with 1 µg/mL anti-σ^T^ in 3% milk powder in TBS-Tween. Goat anti‐rabbit horseradish peroxidase (HRP)‐conjugated secondary antibodies (Invitrogen #32460) with a 1:5,000 dilution was allowed to bind for 4 h. Bands were visualized using SuperSignal Femto West and an LI-Cor Odyssey Fc system, and relative signal intensities, quantified using the Image studio software (4.0).

### Statistical analysis

Simple linear regression analysis in GraphPad Prism (v10.0.2) was used to test whether the decrease of σ^T^ observed in *in vitro* degradations with and without ATP and the corresponding regeneration system (Fig. 2B, +ATP and –ATP, respectively) are different. The options used were as follows: (i) test whether slopes and intercepts are significantly different, (ii) consider each replicate Y value as an individual point, (iii) start regression line: auto, and (iv) end regression line: auto. To assess if differences in *in vivo* σ^T^ levels were statistically significant (Fig. 3E; Fig. 5B), multiple unpaired *t*-test analyses in GraphPad Prism (v10.0.2) were used. The values of each time point were compared individually using a Welch *t*-test assuming Gaussian distribution and without an assumption about consistent standard deviations. As the values of each time point of each condition were compared only once with the corresponding values of the other condition, no *P*-value correction was performed. In addition, the comparison of time point −60 (pre-stress) (Fig. 3E) was ignored, as the quantification of each experiment was normalized to that timepoint of the wild type resulting in an SD of 0.

## Data Availability

All relevant data supporting the findings of this study are provided in the main figures or supplemental figures. The study makes use of mass spectrometry proteomics data sets described in references 10 and 20. The Lon^Trap^ proteomics data (20) have been deposited to the ProteomeXchange Consortium via the PRIDE (28) partner repository with the database identifier PXD036514.

## SUPPLEMENTAL MATERIAL

Supplemental figures and tables jb.00228-23-s0001.pdf Figures S1 and S2 with figure legends and Tables S1 and S2.

ASM does not own the copyrights to Supplemental Material that may be linked to, or accessed through, an article. The authors have granted ASM a non-exclusive, world-wide license to publish the Supplemental Material files. Please contact the corresponding author directly for reuse.
