# Supplementary material for: Regulation of the general stress response sigma factor σT by Lon-mediated proteolysis
Source: J Bacteriol. 2023 Nov 6;205(11):e00228-23. doi: 10.1128/jb.00228-23 (PMC10662116; doi:10.1128/jb.00228-23)
Supplement: Supplemental figures and tables — Figures S1 and S2 with figure legends and Tables S1 and S2. [file jb.00228-23-s0001.pdf]

Supplemental Material for:

Regulation of the general stress response sigma factor σ^T by Lon-mediated proteolysis

Roya Akar[1], Matthias J. Fink[1], Deike J. Omnus[1] and Kristina Jonas[1]*

Science for Life Laboratory and Department of Molecular Biosciences, The Wenner-Gren Institute, Stockholm University, Svante Arrhenius väg 20C, Stockholm 10691, Sweden

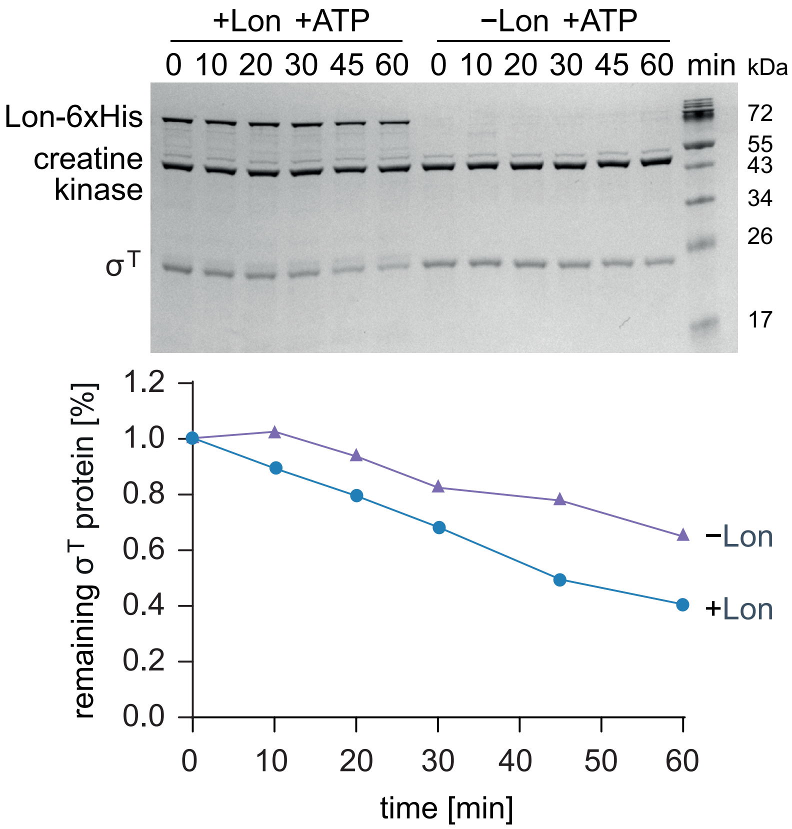

Figure S1. *In vitro* σ^T stability in the absence and in the presence of Lon.
In vitro degradation assays showing changes in σ^T levels over 60 min, either in the presence of Lon (+Lon), or in the absence of Lon (−Lon). Both reactions contained ATP and creatine kinase for ATP regeneration. The graph shows quantifications of σ^T band intensities (normalized to creatine kinase) on the gel shown above.

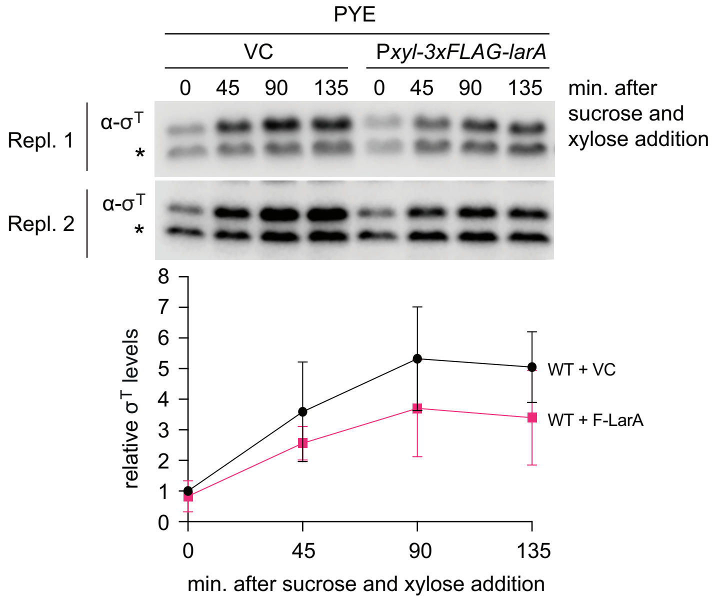

Figure S2. Effect of *larA* overexpression on σ^{T} during sucrose treatment in cultures grown in PYE medium.

Immunoblot analysis of σ^{T} abundance in *C. crescentus* wild type (WT) cells harboring an empty vector (VC) or a vector for xylose-inducible *larA* expression (P_{xyl}-*3xFLAG-larA*) before (t=0 min) and after 45, 90 and 135 min exposure to xylose and 150 mM sucrose in PYE + gentamycin medium. Two biological replicates are shown (Repl. 1, Repl. 2). Quantifications below the immunoblot display means ± SD of the two biological replicates.

Table S1. Bacterial strains used in this study

Strain	Description	Reference
NA1000 (CB15N)	Synchronizable *C. crescentus* wild type	(1)
KJ546	*C. crescentus* Δ*lon* (*lon*::Ω re-introduced into NA1000 by phage transduction)	Provided by M.T. Laub
ML161	*C. crescentus* Δ*sigT*	(2)
BL21-SI	*E. coli*, salt inducible version of BL21	Provided by Claes Andréasson
Dh5α	*E. coli* cloning strain	Invitrogen

Table S2. Plasmids used in this study.

Name	Description	Marker	Reference
pDJO307	pBX-MCS-4 containing P_{xyl}-*3xFLAG-larA*	$gent^R$	(3)
pSUMO-YHRC	Plasmid for protein expression using PT7 with an N-terminal 6xHis-SUMO tag.	kan^R	(4) RRID:Addgene_54336
pMF59	pSUMO-YHRC containing *6×His-SUMO-sigT*	kan^R	This study

Supplemental Material References

1. **Evinger M, Agabian N.** 1977. Envelope-associated nucleoid from *Caulobacter crescentus* stalked and swarmer cells. J Bacteriol **132:**294-301.
2. **Alvarez-Martinez CE, Lourenco RF, Baldini RL, Laub MT, Gomes SL.** 2007. The ECF sigma factor sigma(T) is involved in osmotic and oxidative stress responses in *Caulobacter crescentus*. Mol Microbiol **66:**1240-1255.
3. **Omnus DJ, Fink MJ, Akar R, Kallazhi A, Jonas K.** 2022. The heat shock protein LarA activates the Lon protease at the onset of proteotoxic stress doi:https://doi.org/10.1101/2022.10.10.511565, BioRxiv.
4. **Holmberg MA, Gowda NK, Andreasson C.** 2014. A versatile bacterial expression vector designed for single-step cloning of multiple DNA fragments using homologous recombination. Protein Expr Purif **98:**38-45.
